# Supplementary material for: Charge transfer from the carotenoid can quench chlorophyll excitation in antenna complexes of plants
Source: Nat Commun. 2020 Jan 31;11:662. doi: 10.1038/s41467-020-14488-6 (PMC6994720; doi:10.1038/s41467-020-14488-6)
Supplement: Supplementary file 1 — Supplementary Information [file 41467_2020_14488_MOESM1_ESM.pdf]

Charge transfer from the carotenoid can quench chlorophyll excitation in antenna complexes of plants

Supplementary Information

Lorenzo Cupellini[1]*, Dario Calvani[1], Denis Jacquemin[2], Benedetta Mennucci[1]*

[1] *Università di Pisa, Dipartimento di Chimica e Chimica Industriale, Via G. Moruzzi 13, 56124 Pisa (PI), Italy*

[2] *Laboratoire CEISAM-UMR CNRS 6230, Université de Nantes, 2 Rue de la Houssiniere, BP-92208,F-44322 Cedex 3 Nantes, France*

* Corresponding author. e-mail: LC lorenzo.cupellini@unipi.it BM benedetta.mennucci@unipi.it

Supplementary Methods

Molecular dynamics

The preparation and setup of the MD is described in a previous work by some of us.[1] Initially, the crystal structure of trimeric spinach LHCII (PDB code: 1RWT, chains C,H,E)[2] is placed in a DOPC phospholipid membrane with 450 lipids per layer. On the one hand, this is a simplified description of the thylakoid membrane, which is instead a mixture of several non-common lipids. On the other hand, many experiments have been conducted in non-native environments, and only recently there has been a spectroscopic comparison between native-like and detergent environments for LHCII.[3] Our strategy of using a model membrane is consistent with the work of Liguori *et al.*[4].

The MD protocol has been adapted from the simulation of Photosystem II by Ogata *et al.*:[5]

1. The minimization is performed in three steps: (i) minimization of all hydrogen atoms, (ii) minimization of all added ions and water molecules, (iii) full minimization of the system. All minimizations are performed with 500 steps of steepest descent followed by 500 steps of conjugate gradient;

2. The system is heated to 300K in the NVT ensemble for 30 ps. The protein, all cofactors, and all crystallographic waters are restrained by a harmonic potential with a 5 KCal mol^{-1}Å^{-2} force constant;

3. The system is equilibrated at 300K in the NPT ensemble with anisotropic pressure coupling. The same restraints used in the heating are initially set, and next released over 200 ps, reducing the force constant by 0.25 KCal mol^{-1}Å^{-2} every 10 ps. The equilibration is completed with an additional 2 ns run without constraints.

4. The production run (2.779 μs) is performed in the NPT ensemble at 300K, without any constraint. Snapshots are saved every 100 ps.

All MD runs are performed with the Amber14 suite of programs, using the GPU version of *pmemd*. The SHAKE algorithm is used only in the production run in order to allow for an integration time step of 2 fs. The Langevin thermostat is used, with a collision frequency of 1 ps^{-1}, and for the NPT runs the Berendsen weak-coupling algorithm is used with a coupling constant of 1 ps^{-1}. The Amber ff14SB force field is used for the protein. Carotenoids are modeled with an *ad hoc* force field developed by Prandi *et al.*,[6] and chlorophyll parameters are taken from the literature.[7] The lipid14 force field[8] is used for the membrane lipids, whereas the lipid11 force field is used for the internal DPPG. Water molecules are described with the TIP3P model.

Analysis of the microsecond MD trajectory.

The differences between the MD structure and the crystal structure is analyzed by plotting the RMSD to crystal for each of the domains of LHCII. As shown in Figure 1, the transmembrane helices (A,B,C) remain very close to the crystal, with RMSD values well below 2Å, whereas only Helix D is more mobile. Stromal and lumenal loops also remain close to the crystal, whereas the C-terminus is very flexible. In contrast to the monomer simulation by Liguori *et al.*,[4] the C-terminus is much more flexible in our case, whereas the N-terminus remains relatively close to

the crystal conformation. This is probably due to the interaction between the monomers in the trimeric structure (See Figure 1b of the main text) where the N-termini interact with the helix C of the other monomers.

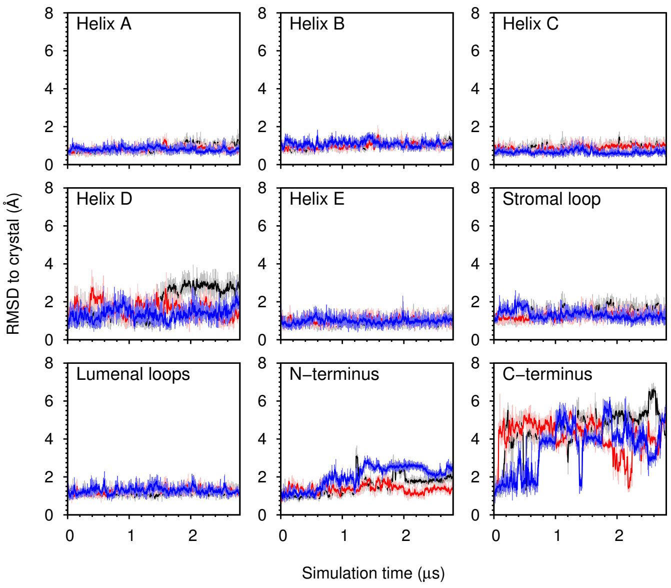

Supplementary Figure 1: RMSD of the protein domains along the MD trajectory. The three colours represent the three different monomers (black, M1; red, M2; blue, M3). The y-axis scale is the same as in Ref. 4.

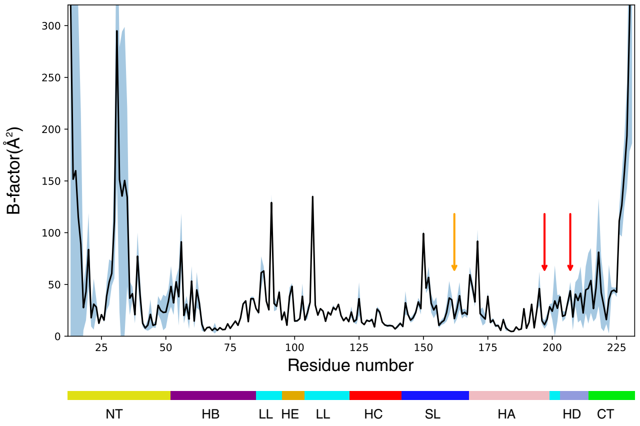

Supplementary Figure 2: B-factor of the protein domains in the MD trajectory (average of the three monomers). The average of the B-factors of all atoms in each residue is shown (*i.e.*, including sidechains). The shaded area represents twice the standard error of the mean computed over the three monomers. The vertical arrows approximately correspond to the binding sites of Lut1. The different domains are highlighted at the bottom of the plot (NT, N-terminus; HB, Helix B; LL, Lumenal Loop; HE, Helix E; HC, Helix C; SL; Stromal Loop; HA, Helix A; HD, Helix D; CT, C-terminus), color-coded as in Figure 1 of the main text.

We also analyze the torsional angle between helices A and D as suggested by Daskalakis et al.[9]

by computing the ϕ Ramachandran angle of Gly204. The results, reported in Figure 3, show that helix D does not change conformation with respect to the crystal structure. This is consistent with our LHCII model remaining close to the "quenched" conformation.

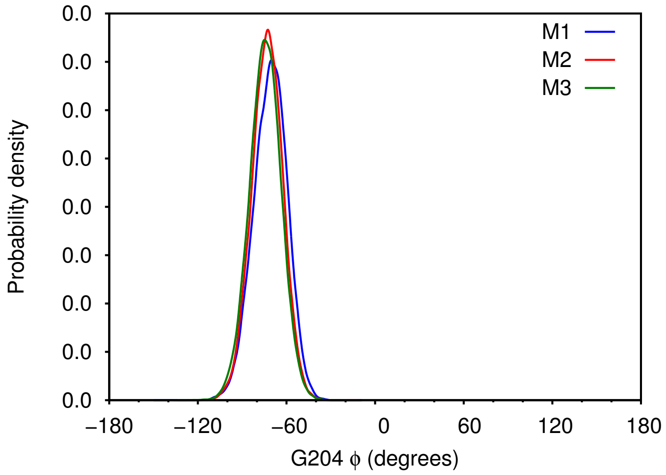

Supplementary Figure 3: Distribution of the ϕ Ramachandran angle of Gly204 in the three monomers.

Benchmark of charge transfer states

The difficulties of TD-DFT, with or without the application of the Tamm-Dancoff approximation, in describing on an equal footing both CT and local excited states are well known.[10–12] In order for TD(A)-DFT to reproduce the correct $\frac{1}{R}$ asymptotic behaviour of a long-range CT, it is necessary to introduce exact exchange in the DFT functional. Long-range corrected functionals can recover such a correct asymptotic behaviour, even exactly when the contribution of exact exchange becomes 100% at long interelectronic distance.[13–16] Whilst such long-range corrected functionals do provide accurate CT transition energies, they tend to overestimate the transition energy of local excited states.[17]

In order to appraise the quality of our level of theory, we benchmarked the excitation energies of Lut-Chl dimers using second-order wavefunction approaches. More in details, we performed second-order coupled-cluster (CC2) and Algebraic Diagrammatic Construction [ADC(2)] calculations on four Lut-Chl dimer structures extracted from the 240 structures used in DFT calculations. These ADC(2) and CC2 calculations have been performed with the Turbomole program,[18] applying the RI approximation and selecting def2-SV(P) basis set. Such choice is dictated by the size of the investigated dimers (171 atoms). The comparisons are reported in Table 1 considering different key excited states. Globally, one notices that the ADC(2) transition energies are slightly smaller than their CC2 counterparts, especially for the low-lying LE transitions, which is a typical outcome for transitions close or below than 2 eV.[19] We therefore consider the CC2 values as reference here. For the CT state, we find that the TDA/ωB97X-D values agree quite well with the CC2 results, with a mean/maximum deviation of 0.096/0.14 eV, which is significantly better than the expected accuracy of TD-DFT (ca. 0.25 eV). In contrast, the ωB97X functional overestimates the CT energies by ~0.5 eV, a quire large value, which is probably the consequence of the larger

attenuation parameter in ωB97X than ωB97X-D; the former functional presenting a steeper onset of exact exchange.[16,20]

Supplementary Table 1: Comparison of the LE (Q_y) and CT energies in selected Lut-Chl dimer structures.

State	TDA/ωB97X	TDA/ωB97X-D	ADC(2)	CC2
		Dimer A		
LE	2.18	2.19	1.97	2.18
CT	3.46	3.04	2.90	2.96
CT-LE	1.28	0.85	0.93	0.78
		Dimer B		
LE	2.13	2.15	1.86	2.13
CT	3.09	2.71	2.59	2.65
CT-LE	0.96	0.56	0.73	0.52
		Dimer C		
LE	2.22	2.22	1.91	2.16
CT	3.28	2.88	2.70	2.77
CT-LE	1.06	0.66	0.79	0.61
		Dimer D		
LE	2.13	2.16	2.01	2.19
CT	3.40	2.99	2.83	2.85
CT-LE	1.27	0.83	0.82	0.66

Analysis of charge-transfer energies

CT energies in vacuo. In order to disentangle the effects of internal geometry of the dimer from the effects of the environment on the CT transitions energies, we use calculations *in vacuo* for dimers *a612*/Lut1 and *a603*/Lut2 of monomer 2 along 80 frames of the trajectory. These calculations allow to test which geometrical features contribute the most to the variability of the CT energy. A multivariate linear regression is performed in order to assess the influence of various structural parameters on the CT energy. The choice of geometrical parameters is based on physical reasoning. The energy of a $D \to A$ CT transition depends on the HOMO/LUMO difference as well as on the distance between the donor and acceptor:

$$E_{\mathrm{CT}} \simeq E^{A}_{\mathrm{LUMO}} - E^{D}_{\mathrm{HOMO}} - f(R) \tag{S1}$$

for large R, clearly $f(R) \to 1/R$; however, for close separations, the exact functional dependence is more complicated than a simple inverse function with the center/center distance. The E^{A}_{LUMO} and E^{D}_{HOMO} energies, as a first approximation, mainly depend on internal DOFs of each chromophore. Therefore, we need to include both intra-chromophore and inter-chromophore coordinates in order

to explain geometrically the variability of CT energies.

We define two internal coordinates for each chromophore (Lut and Chla), based on the bond length alternation (BLA) between single and double bonds along the conjugation paths highlighted in Figure 3a of the main text. The BLA is defined as

$$\text{BLA} = \frac{1}{n_{\text{single}}}\sum_{i}^{\text{single}} l_i - \frac{1}{n_{\text{double}}}\sum_{j}^{\text{double}} l_j$$

where $n_{\text{single/double}}$ are the number of single and double bonds, and $l_{i/j}$ are the lengths of single and double bonds, respectively.

In order to capture the intermolecular dependence of the CT energy, we define the density overlap[21] between the atoms in Lut and Chla highlighted in Figure 3b of the main text. This overlap is the integral of a product of spherical densities centered on the atoms of the two molecules:

$$\text{OVLP} = \sum_{i\in\text{Chla}}\sum_{j\in\text{Lut}} \frac{1}{(2\pi(\sigma_i^2+\sigma_j^2))^{\frac{3}{2}}} \cdot e^{\frac{(\vec{r}_i-\vec{r}_j)^2}{2(\sigma_i^2+\sigma_j^2)}}$$

where $\vec{r}_i$ and $\vec{r}_j$ are the positions of atoms i and j, and σ_i and σ_j are the respective van der Waals radii. Finally, we take the position (x, y, z) of the lutein center on a reference frame defined by the chlorin plane of the Chla.[1]

The internal BLA coordinates considered alone explain more than 50% of the variability of the CT energy *in vacuo* and already give a good correlation with the QM calculations (See Figure 4). Inclusion of the intermolecular coordinates allows to explain more than 70% of the total variance of the CT energy (Figure 4, blue points). As this model includes the main intermolecular degrees of freedom, the unexplained variability originates from other intramolecular coordinates of Lut and Chla.

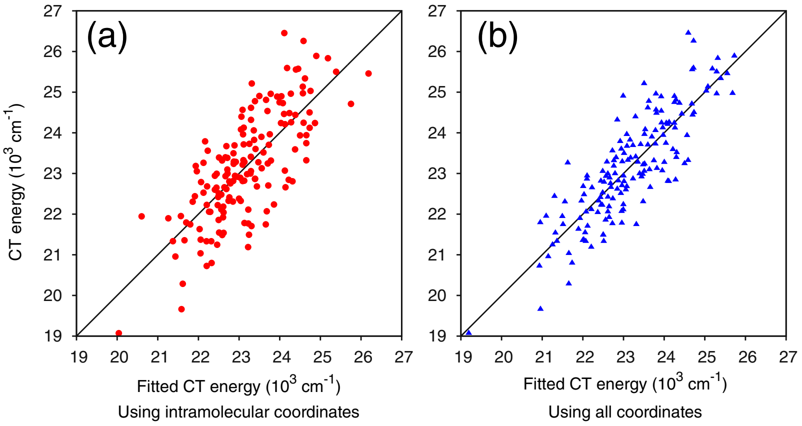

Supplementary Figure 4: Comparison between the CT energy calculated *in vacuo* on each frame, and the CT energy predicted on the basis of (a) only the internal coordinates of both pigments ($R^2 = 0.55$), or (b) all coordinates ($R^2 = 0.72$).

The above analysis shows that the CT energy is mainly modulated by the internal coordinates Lut and Chla, respectively through the HOMO and LUMO. From the regression parameters (Table 4) we estimate that an increase of 1×10^{-2}Å^3 in the overlap would result in a decrease of

~400 cm^{-1} in the CT energy.

Effect of the environment on the CT energy. In Figure 5 we report the distributions of the environment shift on the CT energy (MMPol minus vacuum), for sites L1 and L2. If both distributions are broad, spanning a few thousands of cm^{-1}, there is a significant difference (320 ± 120 cm^{-1}) between L2 and L1.

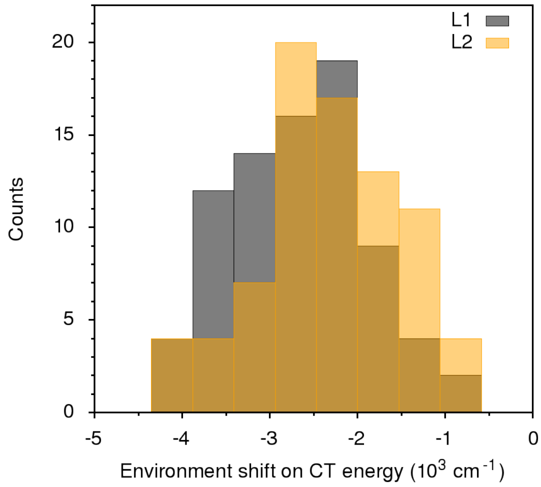

Supplementary Figure 5: Histogram of the environment-induced shift of the CT energy for sites L1 and L2.

Inter-chlorophyll charge transfer states

Supplementary Table 2: Average values and 95% confidence intervals of energies (E) and couplings (V) in the Chl-Chl dimers. Coupling averages are given as root-mean-square (RMS) instead of arithmetic mean. Reorganization energies (λ) and driving forces (ΔG) are also given, as estimated from the variance of the CT and LE energies. All values in cm^{-1}. CT always indicates the lowest CT state of the dimer.

Parameter	*a612*/*a611* (L1)		*a603*/*b609* (L2)	
CT state	$a612^+a611^-$		$a603^+b609^-$	
E(Chl*) Q_y	15 587	±72	15 548	±74
E(CT)	22 276	±	20 707	±74
V(Q_y,Q_y)	232	±4	149	±5
V(Q_y,CT)	379	±27	250	±25
$\lambda_{\text{CT-LE}}$ [a]	6 198		5 168	
$\Delta G_{\text{CT-LE}}$ [a]	1 709		871	

Dependence of LHCII lifetime on the CT energetics

We show in Figure 6 how the lifetime of LHCII depends on the driving force of charge separation in site L1. The shaded region represents the variation of the LHCII lifetime obtained by doubling or halving the charge-recombination rate to the GS. For the value computed in the present work ($\sim$ -80 cm^{-1}) we obtain a lifetime at the lower bound of the lifetimes for crystal LHCII (0.3–0.7 ns), whereas for $\Delta G \gtrsim 700\ \mathrm{cm}^{-1}$ we obtain values compatible with LHCII in the photosynthetic membrane.[22] Note that this analysis assumes a fixed value for the CT coupling and for all parameters of the L2 site.

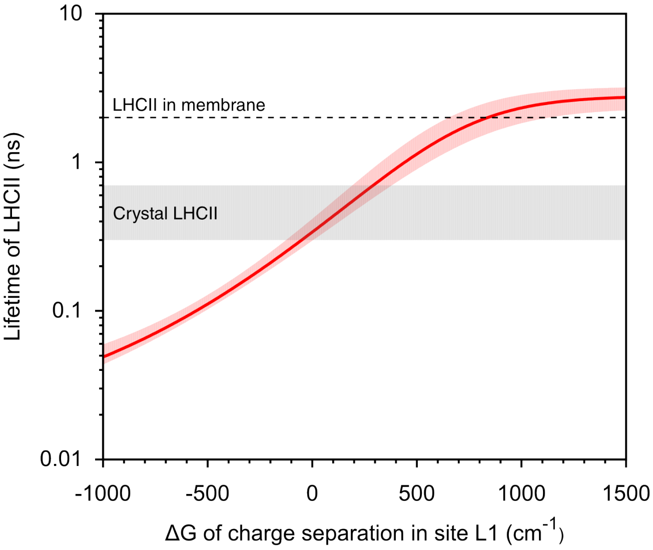

Supplementary Figure 6: Dependence of the LHCII lifetime on the free energy of charge separation in the L1 site (red line). The parameters of the kinetic model are the same as in Figure 2b of the main text, except for the forward and reverse charge-separation rates for the *a612*-Lut1 dimer, which are both determined by ΔG on the x-axis. The shaded region corresponds to varying the rate for charge-recombination to the ground state from 20 ps (upper bound) to 5 ps (lower bound).

Supplementary Figures

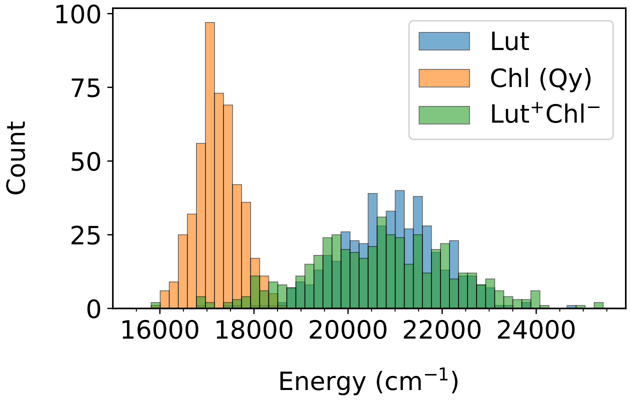

Supplementary Figure 7: Histograms of excitation energies for all the QM/MMPol calculations in the present work.

Supplementary Tables

Supplementary Table 3: CT energies and couplings computed on the crystal structure

	CT energy (cm^{-1})		CT coupling (cm^{-1})	
Site	L1	L2	L1	L2
Monomer 1	21470	24306	47	236
Monomer 2	21473	24864	9	152
Monomer 3	20845	23620	58	143

Supplementary Table 4: Coefficients of the linear regression for the CT energy in vacuo. The units of BLA and Overlap are respectively pm and 10^{-2}Å^3, whereas the units of x_{Lut} and y_{Lut} are Å.

Intercept	19031.8	±	2027.5
BLA_1^{Lut}	243.1	±	65.4
BLA_2^{Lut}	202.9	±	41.1
BLA_1^{Chl}	164.3	±	36.1
BLA_2^{Chl}	207.6	±	32.5
Overlap	-392.0	±	105.5
x_{Lut}	290.1	±	118.9
y_{Lut}	-378.1	±	249.3

Supplementary References

[1] Balevičius, V. *et al.* Fine control of chlorophyll-carotenoid interactions defines the functionality of light-harvesting proteins in plants. *Sci. Rep.* **7**, 1–10 (2017).

[2] Liu, Z. *et al.* Crystal structure of spinach major light-harvesting complex at 2.72 Å resolution. *Nature* **428**, 287–292 (2004).

[3] Son, M., Pinnola, A., Gordon, S. C., Bassi, R. & Schlau-Cohen, G. S. Observation of dissipative chlorophyll-to-carotenoid energy transfer in light-harvesting complex ii in membrane nanodiscs Preprint at https://doi.org/10.26434/chemrxiv.8156702.v2 (2019).

[4] Liguori, N., Periole, X., Marrink, S. J. & Croce, R. From light-harvesting to photoprotection: structural basis of the dynamic switch of the major antenna complex of plants (LHCII). *Sci. Rep.* **5**, 15661 (2015).

[5] Ogata, K., Yuki, T., Hatakeyama, M., Uchida, W. & Nakamura, S. All-Atom Molecular Dynamics Simulation of Photosystem II Embedded in Thylakoid Membrane. *J. Am. Chem. Soc.* **135**, 15670–15673 (2013).

[6] Prandi, I. G., Viani, L., Andreussi, O. & Mennucci, B. Combining classical molecular dynamics and quantum mechanical methods for the description of electronic excitations: The case of carotenoids. *J. Comput. Chem.* **37**, 981–991 (2016).

[7] Ceccarelli, M., Procacci, P. & Marchi, M. An ab initio force field for the cofactors of bacterial photosynthesis. *J. Comput. Chem.* **24**, 129–42 (2003).

[8] Dickson, C. J. *et al.* Lipid14: The Amber Lipid Force Field. *J. Chem. Theory Comput.* **10**, 865–879 (2014).

[9] Daskalakis, V. *et al.* Structural Basis for Allosteric Regulation in the Major Antenna Trimer of Photosystem II. *J. Phys. Chem. B* **123**, 9609–9615 (2019).

[10] Dreuw, A., Weisman, J. L. & Head-Gordon, M. Long-range charge-transfer excited states in time-dependent density functional theory require non-local exchange. *J. Chem. Phys.* **119**, 2943–2946 (2003).

[11] Tozer, D. J. Relationship between long-range charge-transfer excitation energy error and integer discontinuity in kohn-sham theory. *J. Chem. Phys.* **119**, 12697–12699 (2003).

[12] Dreuw, A. & Head-Gordon, M. Single-reference ab initio methods for the calculation of excited states of large molecules. *Chem. Rev.* **105**, 4009–37 (2005).

[13] Iikura, H., Tsuneda, T., Yanai, T. & Hirao, K. A long-range correction scheme for generalized-gradient-approximation exchange functionals. *J. Chem. Phys.* **115**, 3540–3544 (2001).

[14] Yanai, T., Tew, D. P. & Handy, N. C. A new hybrid exchange–correlation functional using the Coulomb-attenuating method (CAM-B3LYP). *Chem. Phys. Lett.* **393**, 51–57 (2004).

[15] Vydrov, O. A. & Scuseria, G. E. Assessment of a long-range corrected hybrid functional. *J. Chem. Phys.* **125**, 234109 (2006).

[16] Chai, J.-D. & Head-Gordon, M. Systematic optimization of long-range corrected hybrid density functionals. *J. Chem. Phys.* **128**, 084106 (2008).

[17] Jacquemin, D., Planchat, A., Adamo, C. & Mennucci, B. A td-dft assessment of functionals for optical 0-0 transitions in solvated dyes. *J. Chem. Theory Comput.* **8**, 2359–2372 (2012).

[18] Turbomole v6.6 2014, a development of university of karlsruhe and forschungszentrum karlsruhe gmbh, 1989-2007, turbomole gmbh, since 2007; available from `http://www.turbomole.com` (accessed 13 june 2016).

[19] Jacquemin, D., Duchemin, I. & Blase, X. 0–0 energies using hybrid schemes: Benchmarks of td-dft, cis(d), adc(2), cc2, and bse/gw formalisms for 80 real-life compounds. *J. Chem. Theory Comput.* **11**, 5340–5359 (2015).

[20] Chai, J.-D. & Head-Gordon, M. Long-range corrected hybrid density functionals with damped atom–atom dispersion corrections. *Phys. Chem. Chem. Phys.* **10**, 6615 (2008).

[21] Cupellini, L. *et al.* Coupling to Charge Transfer States is the Key to Modulate the Optical Bands for Efficient Light Harvesting in Purple Bacteria. *J. Phys. Chem. Lett.* **9**, 6892–6899 (2018).

[22] Belgio, E., Johnson, M. P., Jurić, S. & Ruban, A. V. Higher plant photosystem II light-harvesting antenna, not the reaction center, determines the excited-state lifetime - Both the maximum and the nonphotochemically quenched. *Biophys. J.* **102**, 2761–2771 (2012).
